# Supplementary material for: Transcriptome and Metabolome Analysis of Color Changes during Fruit Development of Pepper (Capsicum baccatum)
Source: Int J Mol Sci. 2022 Oct 19;23(20):12524. doi: 10.3390/ijms232012524 (PMC9604368; doi:10.3390/ijms232012524)
Supplement: Supplementary file 1 [file ijms-23-12524-s001.zip › Supplementary Figure_Material.pdf]

Supplementary Material

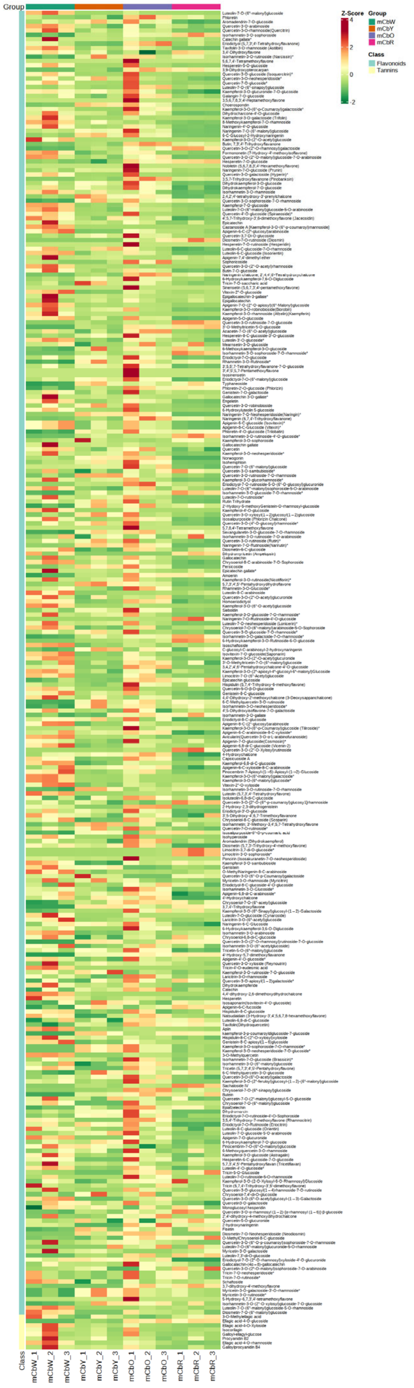

Supplementary Figure S1. Heat map of flavonoid metabolite clustering in three comparative combinations of CbW-vs-CbY, CbY-vs-CbO and CbO-vs-CbR

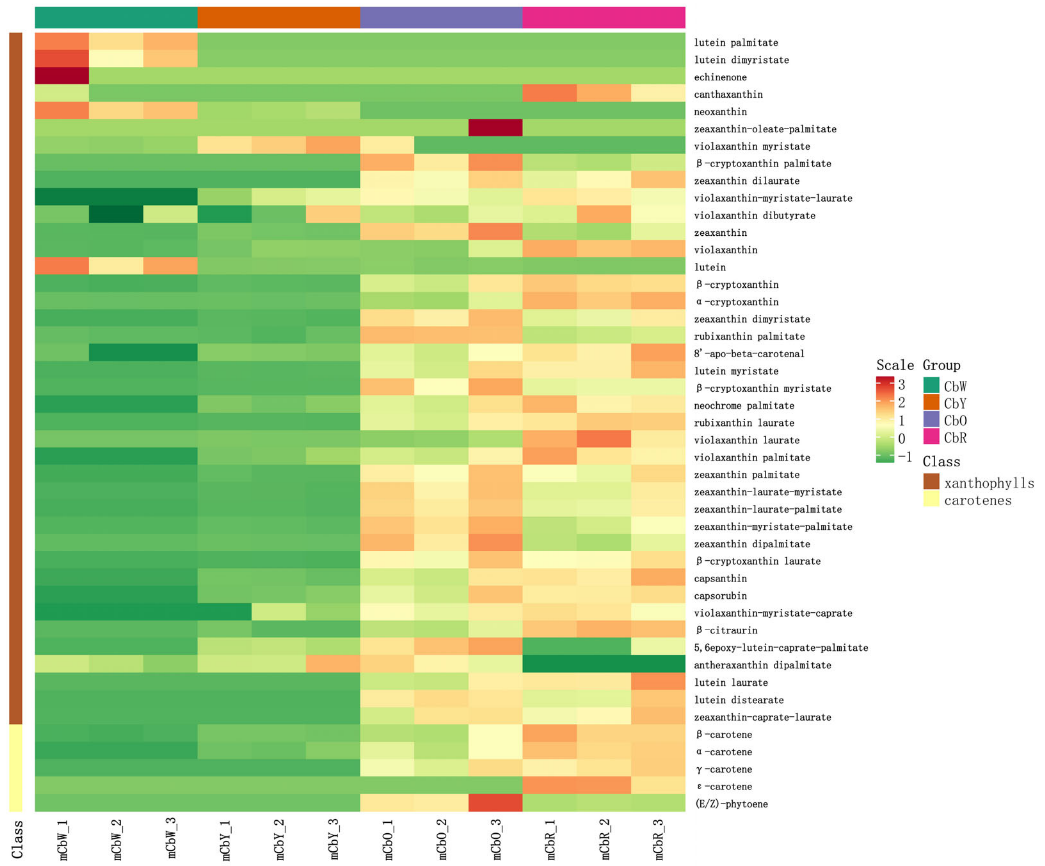

Supplementary Figure S2. Clustering heat map of carotenoid metabolites in three comparative combinations of CbW-vs-CbY, CbY-vs-CbO and CbO-vs-CbR

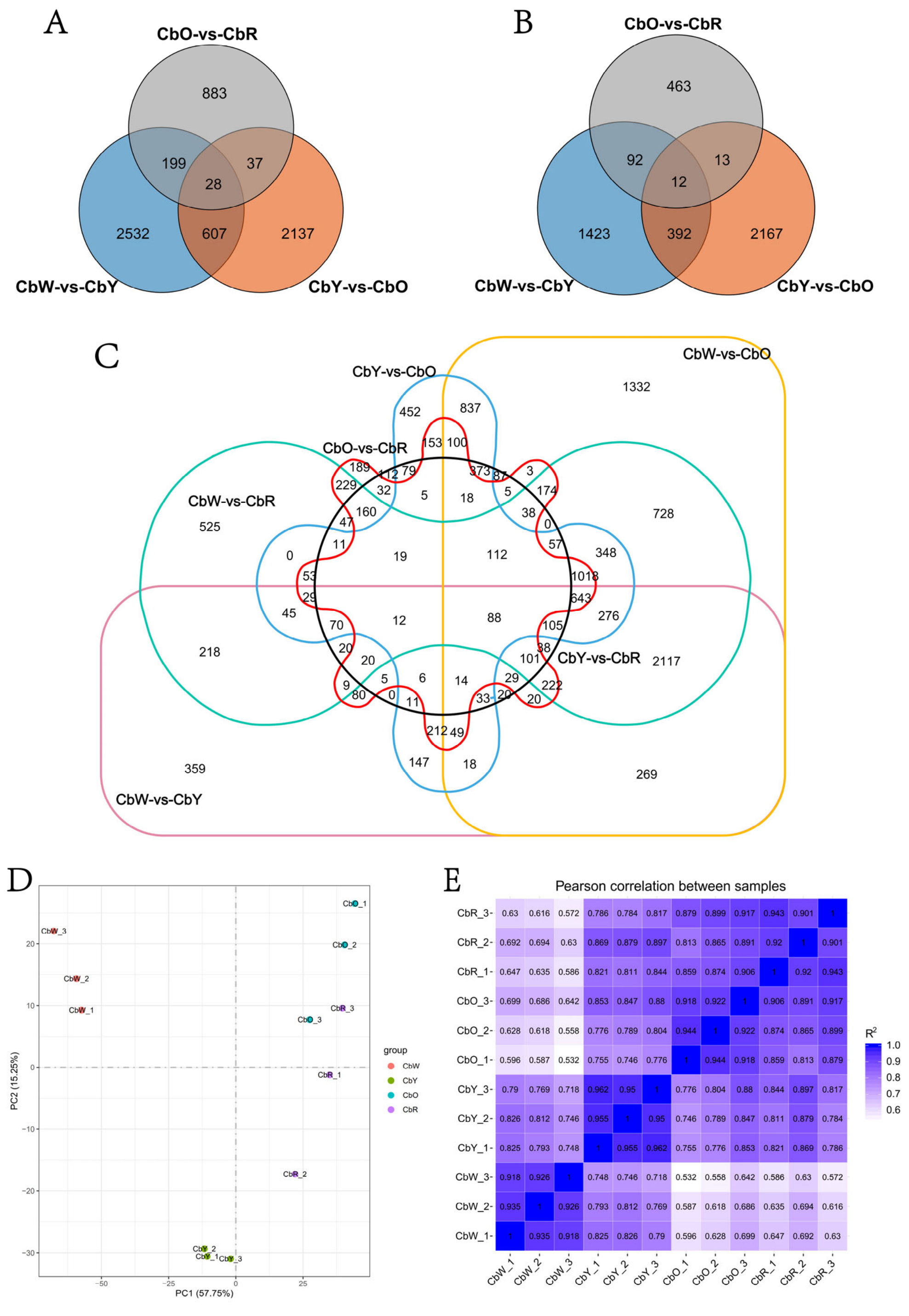

Supplementary Figure S3. Transcriptome analysis. (A) Number of up-regulated DEGs in CbW-vs-CbY, CbY-vs-CbO and CbO-vs-CbR; (B) number of down-regulated DEGs in CbW-vs-CbY, CbY-vs-CbO and CbO-vs-CbR; (C) number of up-regulated DEGs in CbW-vs-CbY, CbW-vs-CbO , CbW-vs-CbR , CbY-vs-CbO, CbY- vs-CbR and CbO-vs-CbR in the total number of DEGs; (D) PCA distribution; (E) sample correlation heat map.

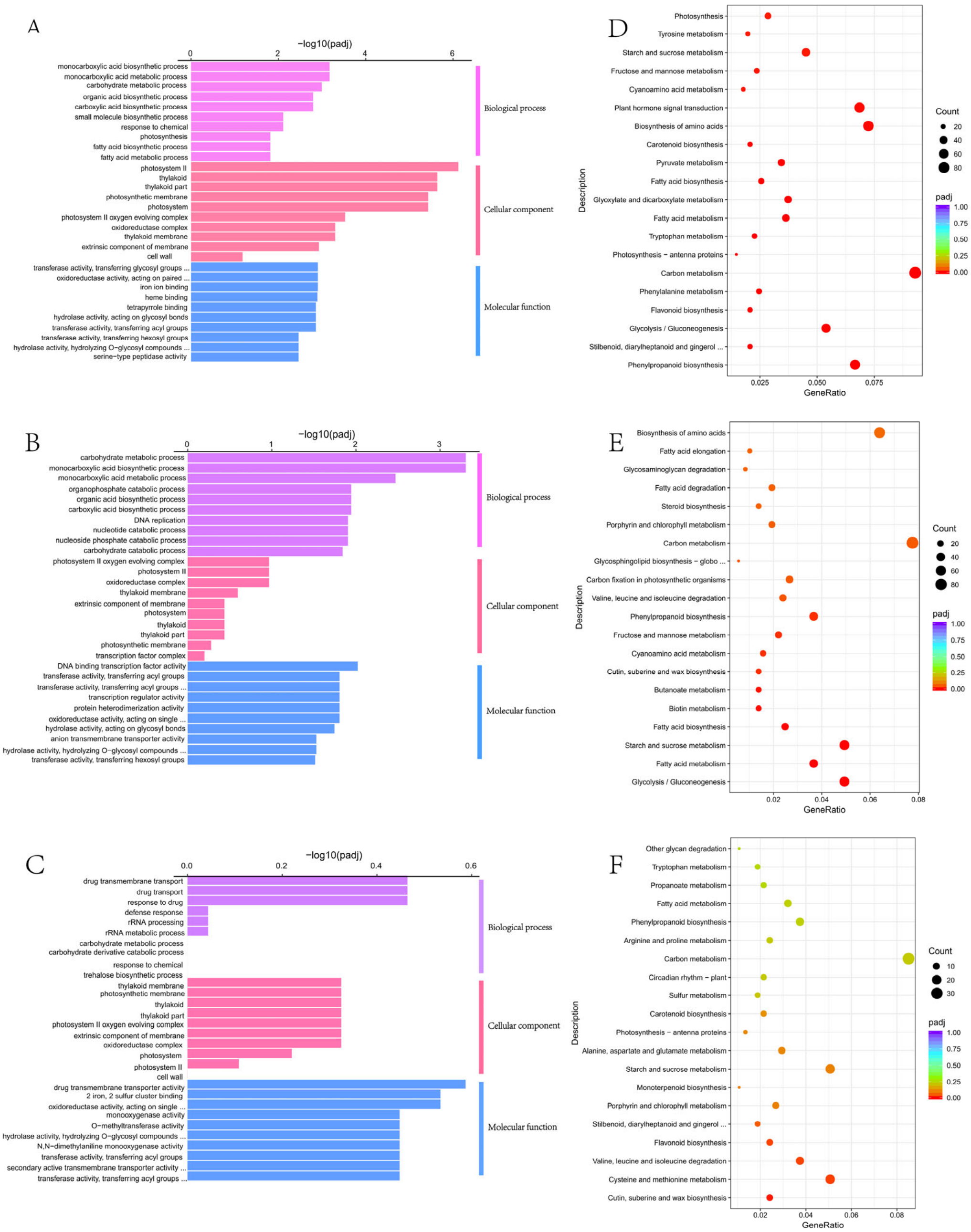

Supplementary Figure S4. GO, KEGG functional enrichment analysis. (A-C) GO functional enrichment for CbW-vs-CbY, CbY-vs-CbO, and CbO-vs-CbR (note: horizontal coordinates are GO Term, vertical coordinates are significance levels for GO Term enrichment, indicated by -log10(padj), different colours indicate different functional classifications, respectively).(D-F) KEGG enrichment analysis of CbW-vs-CbY, CbY-vs-CbO, and CbO-vs-CbR (horizontal coordinate in the figure is the ratio of the number of differential genes annotated to the total number of differential genes on the KEGG pathway, vertical coordinate is the KEGG pathway)

Gene name	CbW-vs-CbY	CbY-vs-CbO	CbO-vs-CbR
CQW23_09483		↓	↑
CQW23_14283	↑		↑
CQW23_07725	↓	↓	↑
CQW23_29956	↑		↑
CQW23_10307	↑		↑
CQW23_30321		↓	↑
CQW23_06623	↑	↑	
CQW23_28374	↑	↑	
CQW23_01678	↑		↓
CQW23_00879	↑	↑	
CQW23_05387	↑	↓	
CQW23_01033	↓	↓	

Supplementary Figure S5. Genes expressed in 2 and 3 combinations during carotenoid synthesis

Gene name	CbW-vs-CbY	CbY-vs-CbO	CbO-vs-CbR
CQW23_24900	↑	↓	
CQW23_19845	↑	↓	
CQW23_32878	↑	↓	

Supplementary Figure S6. Genes expressed in 2 and 3 combinations during flavonoid synthesis
