# Supplementary material for: Transcriptome and Metabolome Analysis of Color Changes during Fruit Development of Pepper (Capsicum baccatum)
Source: Int J Mol Sci. 2022 Oct 19;23(20):12524. doi: 10.3390/ijms232012524 (PMC9604368; doi:10.3390/ijms232012524)
Supplement: Supplementary file 1 [file ijms-23-12524-s001.zip › Supplementary Table S9.pdf]

Supplementary Table S9 Transcriptome sequencing quality analysis

Sample	Raw reads	Clean reads	Total map	Error rate	Q20	Q30	GC%
CbW-1	43339624	41387756	37889927(91.55%)	0.02	98.34	94.82	42.12
CbW-2	41186554	42549580	38926046(91.48%)	0.02	98.41	95	42.19
CbW-3	40288266	40284866	34903530(86.64%)	0.02	98.68	95.67	42.05
CbY-1	45103418	41531132	35532176(85.56%)	0.02	98.32	94.73	42.33
CbY-2	40894570	39294524	27882879(70.96%)	0.02	98.32	94.74	42.29
CbY-3	43796848	39886958	36282137(90.96%)	0.02	98.53	95.29	42
CbO-1	43432696	41415936	39898628(96.34%)	0.02	98.27	94.6	41.86
CbO-2	43864854	39348806	35130573(89.28%)	0.03	97.98	93.74	41.93
CbO-3	41319840	39171874	36816168(93.99%)	0.02	98.19	94.38	42.09
CbR-1	43612032	42718356	40296462(94.33%)	0.02	98.43	95.02	41.99
CbR-2	40967666	39342468	37709807(95.85%)	0.02	98.41	94.9	42.11
CbR-3	41169508	41514282	34344523(82.73%)	0.02	98.18	94.42	41.61
